# Supplementary material for: Antisense Oligonucleotides Used to Identify Telomeric G-Quadruplexes in Metaphase Chromosomes and Fixed Cells by Fluorescence Lifetime Imaging Microscopy of o-BMVC Foci
Source: Molecules. 2020 Sep 7;25(18):4083. doi: 10.3390/molecules25184083 (PMC7570708; doi:10.3390/molecules25184083)
Supplement: Supplementary file 1 [file molecules-25-04083-s001.pdf]

Supplementary Materials

Antisense oligonucleotides used to identify telomeric G-quadruplexes in metaphase chromosomes and fixed cells by fluorescence lifetime imaging microscopy of *o*-BMVC foci

Ting-Yuan Tseng, Shin-Ya Liu, Chiung-Lin Wang, Ta-Chau Chang,*
Institute of Atomic and Molecular Sciences, Academia Sinica, Taipei 106, Taiwan

* Correspondence: tcchang@pub.iams.sinica.edu.tw

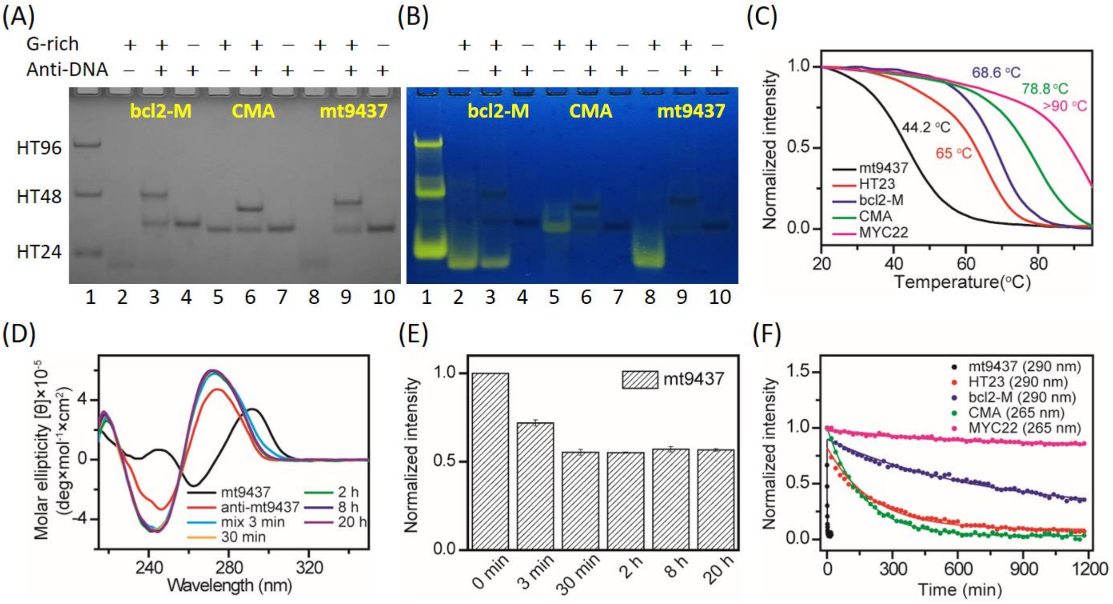

Figure S1. G4 structures unfolded by antisense oligonucleotides in vitro. A) PAGE assays of marker bands of HT24 $(T_2AG_3)_4$, HT48 $(T_2AG_3)_8$, and HT96 $(T_2AG_3)_{16}$ (lane 1) and 40 μM bcl2-M G4 (lane 2), 40 μM bcl2-M G4 after overnight addition of 80 μM anti-bcl2-M (lane 3), 80 μM anti-bcl2-M (lane 4), 40 μM CMA G4 (lane 5), 40 μM CMA G4 after overnight addition of 80 μM anti-CMA (lane 6), 80 μM anti-CMA (lane 7), 40 μM mt9437 G4 (lane 8), 40 μM mt9437 G4 after overnight addition of 80 μM anti-mt9437 (lane 9), 80 μM anti-mt9437 (lane 10). B) Post-stained PAGE assays by 2 μM *o*-BMVC. C) CD melting curves of mt9437, HT23, bcl2-M, CMA, and MYC22 in 100 mM K^+ solution. D) CD spectra of 20 μM mt9437 before and after the addition of 40 μM anti-mt9437 as a function of time. E) The CD intensity normalized to its maximum intensity measured right after the addition of antisense oligonucleotide at 290 nm for mt9437 as a function of time. F) G4 unfolding curves based on the CD intensities at 290 nm of mt9437, HT23, and bcl2-M and at 265 nm of CMA and MYC22 normalized to the intensity measured right after the addition of their antisense sequences as a function of time.
